# Palovarotene (Sohonos), a synthetic retinoid for reducing new heterotopic ossification in fibrodysplasia ossificans progressiva: history, present, and future

**DOI:** 10.1093/jbmrpl/ziae147

**Published:** 2024-11-19

**Authors:** Edward C Hsiao, Maurizio Pacifici

**Affiliations:** Division of Endocrinology and Metabolism, Department of Medicine; the Program in Craniofacial Biology; The Institute for Human Genetics; and The Ely and Edythe Broad Institute for Regeneration Medicine, University of California—San Francisco, San Francisco, CA 94143, United States; Translational Research Program in Pediatric Orthopedics, Division of Orthopaedic Surgery, Children's Hospital of Philadelphia, Philadelphia, PA 19104, United States

**Keywords:** fibrodysplasia ossificans progressiva, retinoids, RARγ, palovarotene, Sohonos, heterotopic ossification

## Abstract

Retinoids are metabolic derivatives of vitamin A and play crucial roles in the regulation of various tissues and organs during prenatal and postnatal development. Active retinoids, like all-trans-retinoic acid, are synthesized in the cytoplasm and subsequently interact with nuclear retinoic acid receptors (RARα, RARβ, and RARγ) to enhance transcription of specific genes. In the absence of retinoids, RARs can still bind to response elements of target genes but repress their transcription. Chondrogenic cell differentiation and cartilage maturation in the growth plate require the absence of retinoid signaling and transcriptional repression by unliganded RARs. This led to the hypothesis that synthetic retinoid agonists may be pharmacological agents to inhibit those cellular processes and counter the excessive formation of cartilage and bone in conditions like heterotopic ossification (HO). HO can be instigated by diverse culprits including trauma, invasive surgeries, inflammatory disorders, or genetic conditions. One such genetic disease is fibrodysplasia ossificans progressiva (FOP), a rare disorder driven by activating mutations in the *ACVR1* gene. Patients with FOP have severe and progressive HO formation in soft tissues, leading to extensive permanent loss of mobility and increased mortality. Synthetic retinoid agonists selective for RARα or RARγ showed efficacy against injury-induced and genetic HO in mouse models. The RARγ agonists showed the highest effectiveness, with palovarotene being selected for clinical trials in patients with FOP. Post hoc analyses of phase II and phase III clinical trials showed that palovarotene has significant disease-modifying effects for FOP, but with significant risks such as premature growth plate closure in some younger subjects. This review provides an overview of retinoid and RAR roles in skeletal development and discusses the identification of palovarotene as a potential FOP therapy, the clinical data supporting its regulatory approval in some countries, and the potential applications of this drug for other relevant disorders besides FOP.

## Introduction

Palovarotene is a synthetic retinoic acid receptor γ (RARγ) agonist that was first described in studies several years ago^1^ and was recently approved for the treatment of fibrodysplasia ossificans progressiva (FOP). FOP is an ultra-rare disease that has a worldwide incidence of about 1 in 1.25-1.36 million livebirths.^2^^,^^3^ It is characterized by formation of extraskeletal endochondral bone at the expense of muscles, connective tissues, and tendons, a process referred to as heterotopic ossification (HO). FOP is caused by activating mutations in the Activin A Receptor, type 1 (*ACVR1*) that encodes the BMP cell surface receptor ALK2.^4^ The mutations—most commonly an R206H substitution—confer on ALK2 a greater basal canonical signaling activity mediated by phosphorylated suppressor of mother against decapentaplegic (SMAD)1/5/8 but also greater responsiveness of protein ligands including various BMPs and activin A.^5–7^ Such enhanced signaling activity is widely thought to provoke the pathological differentiation of local progenitor cells present in muscles and other tissues into cartilage first, followed by endochondral ossification.

The steady accumulation of HO masses causes severe health problems in the patients with FOP, including reduced skeletal mobility and difficulty in respiration and mastication, often leading to premature death.^8^ HO masses cannot be removed by surgery as FOP is a highly reactive disease and any trauma, including surgery, provokes a burst of new HO. For decades, it was realized that drug therapeutics would need to be identified to treat the disease systemically while minimizing any surgical or nonsurgical intervention. Several such treatments are in development in laboratories around the world,^9^ but the first ever to be approved by the United States Food and Drug Administration (FDA) is palovarotene, identified as a potential treatment for FOP in the laboratory of one of us (M.P.).^10^^,^^11^ Subsequent clinical trials carried out by clinical and biomedical groups including those by one of us (E.C.H.)^12^^,^^13^ supported the approval of palovarotene in 2023 for blocking new HO bone formation in patients with FOP. This review charts the history of palovarotene development for FOP therapy in the context of normal retinoid biology in skeletal development and physiology, goes on to illustrate the clinical trials and findings leading to FDA approval, and concludes by delineating future research and clinical goals in this area of biomedical research.

## Vitamin A and RAR genes

Vitamin A is essential for normal embryogenesis, postnatal growth, and systemic health. Vitamin A deficiency causes significant morbidity, including pediatric disorders, organ failures, severe infections, and even early mortality.^14^ The human diet provides a source of compounds that include preformed vitamin A (retinol) as well as provitamin A carotenoids that are converted into vitamin A in the intestine, followed by release into the circulation. The circulating biologically inactive retinol is taken up by cells in given tissues and organs through the cell surface transporter STRAT6^15^ and is then converted into biologically active retinoids—including all-*trans*-retinoic acid (atRA) and 9-*cis*-RA—by the sequential action of cytoplasmic enzymes.^16^ The active retinoids are ferried to the nucleus by binding proteins where they interact with RARs bound to RA response elements (RAREs) on target genes in heterodimeric complexes with retinoid X receptors (RXRs), activating transcription.^17^ In cells lacking active retinoids or rich in CYP26 retinoid catabolic enzymes, the unliganded RAR-RXR complexes bound to RAREs exert transcriptional repression in association with co-repressors such as NCOR1.^18^ Thus, the cellular levels of active retinoids or lack thereof determines whether the RARs act as activators or repressors of target gene transcription, and the balance of transcriptional activation versus repression has critical roles in multiple developmental and physiologic processes.^19^

There are 3 RAR genes in the vertebrate genome and encode RARα, RARβ, and RARγ. The genes are expressed in dynamic and specific spatio-temporal manners prenatally and postnatally. Mouse studies have shown that there is an appreciable degree of functional redundancy amongst them, as indicated by mutants lacking individual RAR genes.^20^ However, mutants lacking both *RARα* and *RARγ* display significant developmental defects that include misshapen vertebral bodies, misaligned and distorted long bones, and digit fusion.^21^ This reaffirms the importance of the RARs in normal development and postnatal health.

## RA and RAR roles in skeletal progenitor cells and cartilage development

It has long been known that mesenchymal progenitor cells emerging in the early embryo undergo a process of condensation and produce initial cartilaginous elements, laying down the overall blueprint and framework of the future skeleton.^22^ Given its fundamental nature, the differentiation of progenitors into cartilage—the process of chondrogenesis—has attracted research interest for decades, and many regulators have been identified including SOX genes and the BMP/transforming growth factor β signaling pathways.^23^ Additional and important regulators of this process are RA and RARs.^10^ Studies using a transgenic *RARE-LacZ* reporter line originally found that mesenchymal condensations undergoing chondrogenesis and cartilage formation lack endogenous active retinoids unlike surrounding retinoid-rich tissues.^24^ Indeed, unliganded RAR repressor function was subsequently shown to be required for chondrogenesis, with RARγ exerting a particularly important role.^25^^,^^26^ In good correlation, the condensed chondrogenic cells were found to strongly express *Cyp26* retinoid catabolic enzymes along with minimal expression levels of retinoid synthesizing enzymes such as *Raldh2*.^25^^,^^26^ Subsequent biochemical and gene expression tests conducted in one of our laboratories (M.P.) showed that the cartilaginous growth plates in growing mice also are largely devoid of endogenous active retinoids and that conditional deletion of *RARγ* along with *RARα* or *RARβ* caused growth retardation and growth plate cartilage tissue disorganization.^27^^,^^28^ These and other studies established the key notion that both chondrogenic cell differentiation and normal growth plate chondrocyte maturation and function require a lack of endogenous active retinoids and unliganded RAR transcriptional repressor function.

## Synthetic RAR agonists and preclinical data for FOP

**Figure 1:**
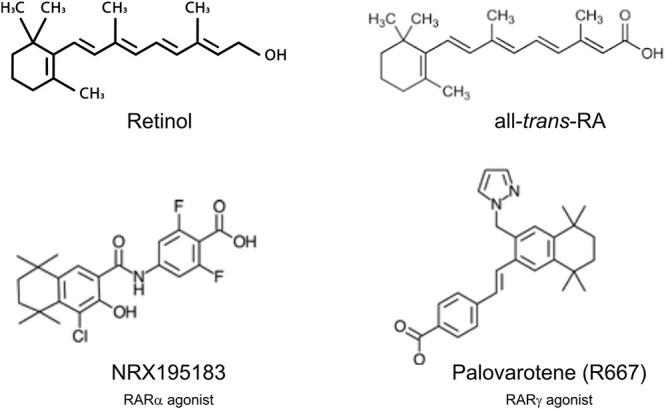
Structures of natural retinoids (top) and synthetic retinoids (bottom).

The key notion above relates quite well with evidence from past studies revealing that exogenous active retinoids are powerful inhibitors of chondrogenesis.^29^^,^^30^ Thus, it became plausible to hypothesize that retinoids could be exploited as therapeutic drugs to inhibit or even prevent the pathological ectopic formation of cartilage and endochondral bone in diseases such as HO^10^. Natural active retinoids such as atRA interact and activate all the RARs (Figure 1), but synthetic retinoid agonists are designed to be selective and have preferential affinity and activity for one RAR^31^^,^^32^ (Figure 1). In initial mouse studies on acquired nongenetic HO, the laboratory of one of us (M.P) reported that a synthetic RARα agonist—NRX195183 (Figure 1)—significantly inhibited formation of heterotopic cartilage and bone in a subdermal mouse model of HO^33^. Building on these findings, it was then found that both RARα and RARγ agonists strongly inhibited experimental HO in mouse models, including genetic HO caused by activating mutations in *ACVR1* as seen in FOP patients.^11^^,^^34^ The RARγ agonists, including palovarotene, displayed much stronger efficacy than RARα agonists, likely reflecting the preponderant roles that the *RARγ* gene has in chondrogenesis and growth plate cartilage.^25^^,^^28^

**Figure 2:**
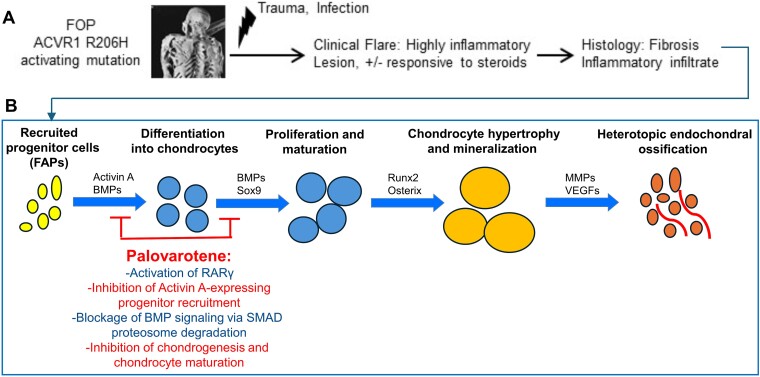
(A) Initial steps leading to induction of heterotopic ossification (HO) including injury/infection and inflammatory infiltrate. (B) Schematic shows the initial chondrogenic and cartilage maturation phases of HO tissue development in fibrodysplasia ossificans progressiva (FOP) and known major regulators including BMPs and activin A; transcription factors Sox9, Runx2, and Osterix; and MMPs and VEGFs. Based on previous studies, palovarotene blocks HO development by inhibiting canonical BMP signaling; recruitment of progenitor cells including those expressing activin A; and chondrogenesis and cartilage maturation. The inhibition of the latter processes in turn inhibits osteogenesis from the cartilage template, thus reducing new HO formation in FOP.

The HO process in FOP patients is induced by flare-ups, injury, infections, or other culprits (Figure 2A) and recapitulates the sequential obligatory steps regulating normal skeletal development (Figure 2B).^35^ It starts with recruitment of progenitor cells at the involved site(s) and is followed by (i) chondrogenesis, (ii) chondrocyte proliferation, maturation, and hypertrophy as normally occurring in the growth plate, and (iii) final replacement of hypertrophic cartilage with endochondral bone (Figure 2B). By binding to RARγ and turning this nuclear receptor into a transcriptional activator, palovarotene counteracts HO by its direct or indirect action on several key developmental steps including (i) inhibition of canonical pSMAD1/5/8 signaling by enhancing SMAD proteosome degradation; (ii) delayed recruitment of skeletal progenitor cells including those expressing Activin A; and (iii) inhibition of chondrogenesis and chondrocyte maturation (Figure 2B).^11^^,^^36^^,^^37^ Given that these developmental steps are obligatory, their inhibition would in turn inhibit chondrocyte hypertrophy and endochondral bone formation, dampening overall HO. Recent studies also suggest that palovarotene can reduce macrophage-driven inflammation *via* inhibition of NFκB.^38^ In sum, the above studies provided the theoretical and factual basis for the repurposing of the RARγ agonist palovarotene for the clinical treatment of HO in FOP patients.^10^

## Clinical studies of palovarotene in pulmonary emphysema

Palovarotene (also known as R-667 or RG-667) was originally developed by Roche Pharmaceuticals and tested in humans as a potential treatment for chronic obstructive pulmonary disease (COPD) and emphysema^1^^,^^39–41^ because retinoid signaling is thought to promote alveologenesis.^42^ Preliminary data in rodents suggested that retinoic acid could mitigate elastase-induced pulmonary emphysema^43^ and that RXR-gamma agonists could reverse the structural, inflammatory, and functional components of cigarette-smoke-induced emphysema.^46^ Early data were collected in Phase I studies in emphysema subjects to look at the pharmacokinetics for palovarotene.^47^

Subjects with α(1) antitrypsin deficiency were used as a model population for the general smoke-induced emphysema population.^48^ Subjects in the REPAIR (Retinoid treatment of Emphysema in Patients in the α(1)-antitrypsin International Registry) study with α(1)-antitrypsin deficiency and emphysema were treated with palovarotene for 1 year at 5 mg/day, with 129 subjects randomly assigned to palovarotene treatment and 133 to placebo. Although palovarotene at this dose was well tolerated, no significant differences in functional lung parameters were identified.^41^ An additional 611 subjects with COPD received multiple doses of palovarotene, in the range 0.2-5 mg/day, for up to 24 months,^1^ also with no significant benefit.

## FOP is a severe debilitating disease of HO

**Figure 3:**
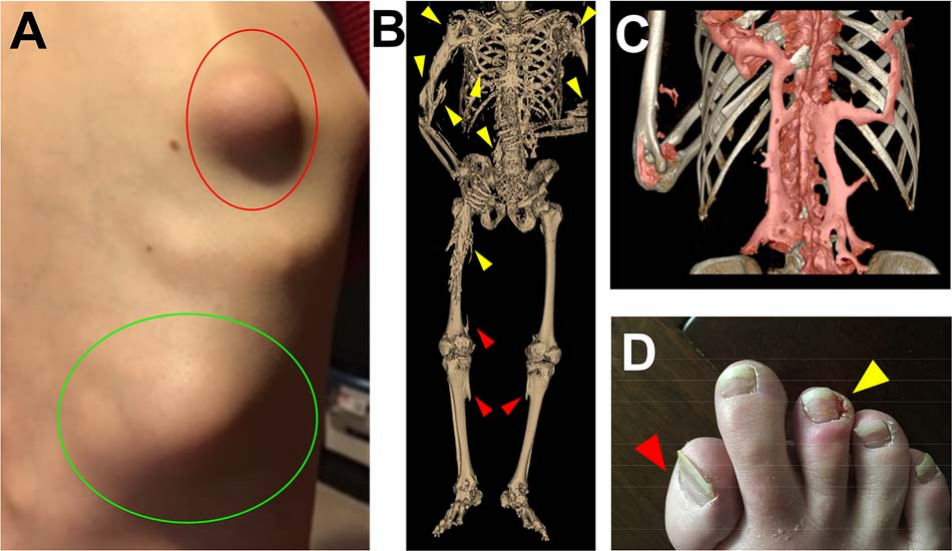
(A) Photograph of a right scapular/back flare, with erythema and induration (indicated by red circle), in a 9-year-old male patient with fibrodysplasia ossificans progressiva (FOP). This flare went on to restrict mobility in the right shoulder. A second older flare, with a different flatter morphology, is also identified (green circle). Image of the top flare was previously published and is reused here under the CC4.0 license.^44^ (B) Low dose whole body CT scan reconstruction is reproduced here^45^ without modification under the CC4.0 license except to add labeling. This 33-year-old male was in the natural history study. Representative osteochondromas are indicated by red arrows. Representative areas of heterotopic ossification (HO) formation are indicated by yellow arrows. (C) False colored thoracic CT scan reconstruction of a 37-year-old female patient with FOP showing accumulated HO (red) overlying the native skeleton. (D) Photograph of a 15-year-old female patient with FOP showing the great toe malformation (shortened, with malformed proximal metatarsal, classically seen in FOP; red arrow) and paronychia (yellow arrow), which can occur in patients taking palovarotene.

FOP is a rare genetic disorder where tissues that are not normally mineralized, such as muscles, tendons, ligaments, and connective tissues, progressively undergo endochondral ossification. This process typically starts early in life and affects almost every joint and skeletal muscle in the body, restricting movement and causing major health problems from an early age^49^ (Figure 3). Over time, the new HO accumulates throughout the body, leading to complete immobility typically by the 20s.^50^^,^^51^ Patients have high morbidity and mortality particularly from the development of thoracic insufficiency syndrome.^8^^,^^52^ The most common cause of death is cardiopulmonary compromise and an inability to clear respiratory infections.^8^^,^^52^^,^^53^

FOP is caused by activating mutations in the *ACVR1* gene that lead to increased canonical signaling by the BMP pathway^4^ and neo-ligand activity by Activin A.^6^^,^^7^ The known mutations in FOP are primarily located in the glycine-serine (GS) region of ALK2, with the most common being the single-point ACVR1 mutation c. G617A leading to a p.R206H substitution.^4^ The latter destabilizes the GS region, causing continuous activation of ALK1. FOP has also been reported in patients with other rare missense mutations in the GS or protein kinase domain of ALK2.^54–57^ All of these gene mutations in FOP disrupt normal homeostasis and cell differentiation processes, triggering the abnormal endochondral ossification that is a hallmark of FOP^58^.

Activins including Activin A normally interact with ALK4 (encoded by *ACVR1B*) and ALK7 (encoded by *ACVR1C*) and activate a canonical signaling pathways mediated by phosphorylated SMAD2/3.^59^ Activin A also interacts with ALK2 and can inhibit canonical pSMAD1/5/9 BMP signaling in some contexts.^6^^,^^60^ In FOP, mutant ALK2 can still interact with Activin A but has been found to trigger signaling by the SMAD1/5/9 pathway, effectively causing a misinterpretation of Activin A as a BMP.^6^^,^^7^ The resulting abnormal activation of ALK2, both through the increased basal signaling activity by BMPs and neo-ligand activity to Activin A, leads to the dramatic flares and bone formation seen in the disease.

Clinically, the most striking initial symptoms of FOP are the massive inflammatory lesions that occur in patients, often with little or no trauma.^61^ These inflammatory flare-ups (Figure 3A), which can be triggered by injuries, infections, or other factors, are associated with increased HO formation (Figure 3B, C), swelling, and severe pain. In addition, FOP is now known to affect multiple organ systems including the cardiopulmonary^8^ and cardiac conduction systems,^52^ the nociceptor and pain sensing pathways,^62^ the immune system,^44^^,^^63^ the renal system via nephrolithiasis,^64^ and the gastrointestinal system as identified in the IFOPA Registry.^65^ Although FOP is clearly a systemic disease, the progressive and unrelenting HO formation leads to the majority of clinical sequala both through direct (eg, nerve stretching around bony growths, leading to pain; HO causing skin erosions) and indirect mechanisms (eg, bowel obstruction due to spinal, abdominal, and chest wall deformities). Thus, reducing or blocking the HO formation particularly in pediatric patients is a critical goal of current therapies in development.

## Palovarotene as a potential therapy for blocking new HO in FOP

**Figure 4:**
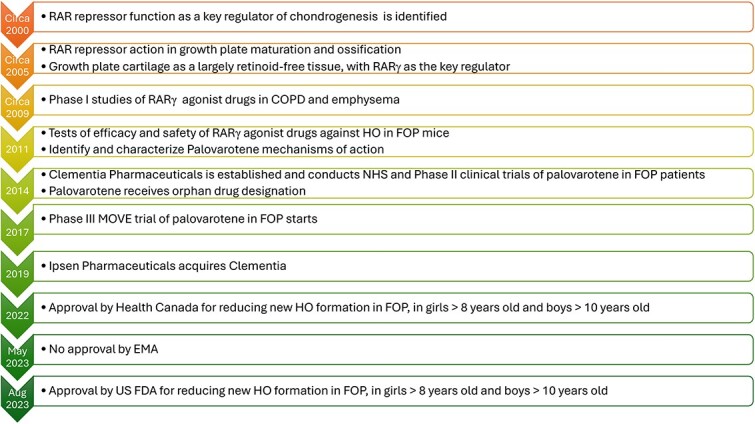
Development timeline of RARγ agonists as a potential therapy for fibrodysplasia ossificans progressiva.

Until recently, corticosteroids and nonsteroidal anti-inflammatory medications (NSAIDs) were the mainstay of flare-up management and prevention of new HO formation, but were not able to prevent HO^53^. Since blocking new HO formation is critical for addressing the major pathologic processes that lead to much of the morbidity and mortality of FOP, palovarotene was studied as a potential therapy for decreasing new HO formation.^10^^,^^11^ Palovarotene was licensed to Clementia Pharmaceuticals in 2014 to develop its use for any clinical indication. Clementia was subsequently acquired by Ipsen Pharmaceuticals in 2019.^66^^,^^67^Figure 4 illustrates the timeline of progression from basic science studies to preclinical mouse studies and clinical trials, culminating with palovarotene approval in the United States in 2023.

## Palovarotene in the FOP phase I and II studies

Palovarotene was studied in a phase I trial in 23 healthy subjects (NCT04829773) to determine pharmacokinetics. Palovarotene was not found to be a significant inducer of Cyp3A4 and showed no adverse events.^68^ As part of the full development program,^69^ palovarotene was first tested in a placebo-controlled double blinded phase II study in subjects with FOP (PVO-1A-201; NCT02190747). Subjects were treated with a flare-based dosing regimen starting within 7 days of flare onset (high dose for 2 weeks), followed by 4 more weeks of low-dose therapy. Weight-based equivalents were used for children.^70^ Subjects were enrolled in 2 cohorts: subjects >15 years old were randomized 3:1 to treatment with palovarotene 10 mg for 2 weeks followed by 5 mg for 4 weeks (abbreviated here as 10/5) vs. placebo, or to a second group of subjects >6 years of age who were randomized 3:3:2 to palovarotene 10/5, palovarotene 5/2.5, or placebo, or a weight adjusted equivalent for skeletally immature subjects. All subjects were allowed standard-of-care therapies including steroids for management of their flare symptoms. Analysis of the pooled data from the 2 arms of the primary endpoint at the flare up site by plain X-ray radiograph at week 6 showed 100% responders with palovarotene 10/5, 88.9% responders with palovarotene 5/2.5, and 88.9% responders with placebo (Cochran–Armitage trend test, *p*=.17). Subsequent follow-up studies at week 12 (6 weeks after the end of palovarotene) showed 95% responders with palovarotene 10/5, 88.9% with palovarotene 5/2.5, and 77.8% with placebo (Cochran–Armitage trend test: *p*=.15). Site-specific CT imaging to quantify new HO volume showed a trend towards reduction with palovarotene treatment but did not meet statistical significance. MRI and ultrasound were also used to assess tissue edema at the site of the flare; however, these did not show significant differences, and practical considerations for these imaging modalities (ie, difficulty positioning subjects in the MRI instrument, limited ability to assess bony structures by MRI) dampened enthusiasm for further use. Palovarotene was well tolerated, and no subjects were discontinued from the study. One subject had a treatment interruption due to elevated serum lipase.

Although these findings were not statistically significant, there was a strong enough trend to support further assessment of palovarotene in a phase III study. In addition, the results and animal studies suggested that higher doses and prolonged dosing with a continuous/chronic dose might have benefits. Thus, this was incorporated into the open label extension studies (202C/204) and Phase III study (MOVE).

## Phase III study of palovarotene in FOP

A subsequent study of palovarotene for treatment of FOP was pursued through a single-arm, open-label, phase III trial (MOVE, PVO-1A-301; NCT03312634), which enrolled palovarotene-naive subjects with both the classical ACVR1^R206H^ and other known ACVR1-activating mutations.^13^ Data from the MOVE study were compared with the FOP natural history study (NHS; NCT02322255), which included subjects with FOP treated with standard-of-care therapies such as corticosteroids and NSAIDs. Subjects aged 4 years and older were treated with daily palovarotene of 5 mg (chronic, when without a flare), or with flare dosing (20 mg for 4 weeks starting at the onset of a clinically defined flare, followed by 10 mg for 8 weeks or longer for low dose flare treatment). Dosing was adjusted for weight if skeletally immature. Annualized change in new HO volume was assessed by low dose whole body CT scan.

Based on the prespecified statistical analysis plan using Bayesian compound Poisson model (BCPM) with square root transformation, the study met interim futility criteria at 12 months and dosing was paused in January 2020. Subsequent evaluation by the independent data safety monitoring board (DSMB) revealed several limitations of the BCPM analysis strategy, specifically due to unexpected negative values of HO formation in some subjects and also differences in the CT assessment schedule between the MOVE study (once every 6 months) and the NHS study (once every year). The DSMB allowed the study to continue, and subsequent post hoc analysis using the BCPM approach without square root transformation at the 18-month timepoint showed a 99.4% probability of any reduction in new HO with palovarotene treatment, with a 60% reduction in new HO volume in MOVE subjects vs. NHS participants. Post hoc 18-month interim analyses using BCPM with square-root transformation and data collapse to unify the NHS and MOVE whole body computed tomography (WBCT) schedules also showed reduction in new HO volume in the MOVE subjects.^13^

Additional post hoc analyses were done as part of the FDA Advisory Board review process and presented in the Ipsen and FDA briefing information.^71^ Matched pair analyses of 39 subjects who transitioned from the NHS study to MOVE, and who therefore served as their own controls, showed that the subjects had reduced new HO formation during the palovarotene treatment phase as compared with while they were enrolled in the NHS study. Although new HO formation can decrease with age, the amount of reduction in new HO formation with palovarotene was greater than what would be expected.^50^ Long-term analysis of subjects who were on palovarotene, but then had their treatment interrupted due to the clinical holds and subsequently resumed therapy, also suggested that mean annualized new HO volume per year was decreased while they were on palovarotene (pre-pause mean of 9427 mm^3^/year, *n* = 97 subjects, interruption mean of 20 108 mm^3^/year, *n* = 42 subjects; and post-restart of 7728 mm^3^/year, *n* = 17 subjects). Furthermore, for the 16 subjects who did not restart treatment, there was increased mean annualized new HO formation when off treatment (2347 mm^3^/year on palovarotene vs. 15 574 mm^3^/year off treatment). Although these analyses have not been formally published to date, they support the conclusion that there was likely a decrease in new HO formation in the subjects treated with palovarotene.

Additional data from the Phase II open label extension studies (PVO-1A-202 and PVO-1A-204) were collected for subjects who were continuing treatment from the initial 201 Phase II study. Although the initial treatment regimen in the 202 study (Parts A and B) involved flare-only dosing as in the 201 study, the PVO-1A-202C study mirrored the Phase III treatment regimen and study assessments. Long-term analysis of subjects in PVO-1A-202C also indicated a reduction in new HO formation.^71^ Thus, although the Phase II and Phase III study results were significantly limited by the post hoc nature the analyses, the multiple approaches and ancillary data suggest that palovarotene has benefits for reducing new HO formation in subjects with FOP.

## Adverse events related to palovarotene in FOP

Although short-term treatment with palovarotene, such as in the Phase II study,^70^ appeared to be very well tolerated, more and severe adverse events were evident with the longer term and high dose treatment regimen used in the phase II study. In the MOVE principal safety set,^13^ all 99 subjects treated with palovarotene reported at least 1 treatment-emergent adverse event (TEAE), with 97.0% experiencing retinoid-associated TEAEs that are consistent with class toxicities (Figure 3D). These common adverse events included dry skin (68.7%), lip dryness (46.5%), alopecia (34.3%), drug eruption (28.3%), pruritus (26.3%), and arthralgia (33.3%). Most TEAEs were mild or moderate and could be managed adequately with symptomatic therapy (such as topical emollients or anti-itching medications) and dose adjustments. 9.1% of subjects had a TEAE that led to permanent discontinuation of palovarotene.

The most concerning serious adverse event observed^13^ was premature physeal closure (PPC), seen in 21 out of 57 subjects under 14 years of age.^13^ Despite extensive examination of the data, no specific risk factor or mechanism could be identified in the patients.^71^ Notably, a mouse model with FOP showed growth plate abnormalities likely worsened by subcutaneous injection of palovarotene in the study^72^ rather than the oral administration route used in the original mouse studies^11^ and in the FOP clinical trials. The seriousness of the PPC led to an FDA-issued partial clinical hold of palovarotene dosing for subjects <14 years of age starting in December 2019, and the final requirement restricting palovarotene prescriptions to children with >90% of potential growth remaining (older than age 10 years old in boys and age 8 years old in girls).^71^

As part of the MOVE study, post hoc analysis of the WBCT data using a new computational method^73^ to estimate bone strength in the native skeleton using finite element analysis revealed a weakening of predicted bone strength in the native skeletons, raising the concern of osteoporosis.^13^ Reduced native bone density has been reported in at least 1 subject with FOP^74^, and subjects with FOP can develop fractures of their native skeletons at an unusually early age suggestive of osteoporosis.^74^^,^^75^ The bone CT computational prediction analysis also identified vertebral deformities in subjects with FOP. Although the vertebral deformities were classified as compression fractures based on the BCT protocol, subjects with FOP are known to have malformed vertebrae.^49^^,^^76^ Since this was the first application of the BCT method to subjects with FOP, further investigation about the clinical significance of these bone findings is underway.

Because palovarotene inhibited osteogenesis, there has been concern about the potential impact of palovarotene on fracture healing. One subject with FOP who underwent bilateral femur surgery for fracture still developed new HO despite palovarotene,^74^ although it is unknown if the amount of new HO was reduced by treatment. Furthermore, 1 subject in the phase III MOVE trial showed impaired fracture healing. The fracture subsequently healed after palovarotene was discontinued, and the subject was able to restart palovarotene after healing was complete.^13^

Palovarotene carries black box warnings on the FDA-approved product insert for teratogenicity (class effect of retinoids) and for premature epiphyseal closure.^77^

## Review, approval history, and clinical use of palovarotene

Palovarotene received orphan drug designation in 2014 from the US FDA (Figure 4). Palovarotene was first reviewed and approved by the Health Canada in January 2022. It was subsequently reviewed by the European Medicines Agency in January 2023 and was recommended the refusal of marketing authorization as a treatment for FOP. This was subsequently confirmed in May 2023, with the major concerns being insufficient data and the lack of functional efficacy (ie, in range of motion, physical function, or quality of life assessments) in the setting of modest reductions in new HO as assessed by radiology.^78^ In addition, there was concern that the measured endpoint (reduction in new HO volume) was not a patient-centered outcome measure and is not used routinely in clinical practice, and that other functional outcomes such as pulmonary function were not assessed.

Palovarotene was reviewed by the US FDA in June 2023 and recommended for approval for marketing in the United States.^71^ The discussion raised important considerations about the adequacy of the NHS study as a comparator group for the MOVE subjects, as the cohorts were similar but not completely identical; the MOVE Phase III study was an open label study and not internally controlled; the need for post hoc analyses due to the problems with the pre-specified statistical analysis for the MOVE study; and the modest reduction in new HO but no clear changes in functionality. However, the discussion also highlighted the rarity and severity of FOP, making it almost impractical to repeat a large study; the lack of effective disease-modifying treatments for this devastating disease; and the ongoing need for intermediate therapies while more definitive therapies are being developed.^71^ The FDA advisory committee voted 10 in favor and 4 against the evidence showing palovarotene to be effective for blocking new HO formation in FOP, and voted 11 for and 3 against the evidence showing that the benefits of palovarotene outweighed the risks for treating patients with FOP. Palovarotene was subsequently given marketing approval by the US FDA in August 2023. Palovarotene is also currently available in the United Arab Emirates, which extends approval to treatments that have been approved by another regulator agency.

After the FDA approval, the International Clinical Council on FOP, a consortium of 21 clinicians from around the world with expertise in the management of FOP, released a statement emphasizing the potential risks and benefits for using palovarotene to treat patients with FOP (ICCFOP.org, August 16, 2023). Particular emphasis was noted for the risks in growing children and the unknown effects on growth plate development, with the recommendation to follow current labeling recommendations and age restrictions (ICCFOP.org) and to support further studies to understand the mechanisms behind this severe adverse event.^53^ In addition, potential risks for drug interactions with palovarotene were emphasized, especially the known risks of pseudotumor ceribri with concomitant retinoids and tetracycline class medications^79^ and the high risk of teratogenicity with all retinoid medications. Long-term studies regarding the safety and efficacy of palovarotene in the real-world setting are still needed.

## Ongoing studies in palovarotene and potential applications outside of FOP

Since the approval of palovarotene by the US FDA in August 2023, several studies continue to follow the course of patients treated with the drug and to better understand the risks and potential use of palovarotene in FOP. The mechanisms for the palovarotene-associated premature epiphyseal closure remain unknown, particularly because closure affects some but not all growth plates. Factors that might cause this complication are also unknown, such as potential links to flare frequency, mutant ALK2 signaling activity levels in the growth plates, or HO tissue accidentally interfering with growth plate activity. Follow-up of the patients affected by PPC in the MOVE study is ongoing. In addition, an open label rollover study for patients >14 years of age (PIVOINE; NCT05027802) is ongoing. More detailed data analyses for the risks of bone disease and osteoporosis, originally identified through the computational analysis of the MOVE and NHS WBCT images, are underway. Finally, the FOPal registry (NCT06089616) has been started and will collect long-term information about safety and efficacy in patients taking palovarotene.

Because palovarotene acts at specific steps during the HO formation process (Figure 2), there has been great interest in whether palovarotene could block other forms of abnormal bone formation besides FOP. Palovarotene showed promise in animal models for hereditary multiple exostoses (HME),^80^^,^^81^ as well as the inhibition of osteochondroma^82^ and chondrosarcoma cells.^83^ Unfortunately, the Phase II study for palovarotene in HME was discontinued due to poor enrollment secondary to the SARS-CoV2 pandemic and the concerns about early epiphyseal closure identified in patients with FOP (NCT03442985).

Recently, there has been interest in examining if the BMP-blocking activity of palovarotene would have benefit for conditions of elevated BMP signaling. Mice with X-linked hypophosphatemia develop mineralization of the bone-tendon attachment site (enthesopathy) in a process driven by BMPs and Indian hedgehog signaling. Treatment with palovarotene attenuated BMP signaling in entheses of Hyp mice, reducing enthesopathy.^84^ In addition, palovarotene may have activity in prostate-cancer-induced bone formation by blocking the endothelial to osteoblast transition that occurs secondary to tumor production of BMP4.^85^

Palovarotene is also now being investigated in several different conditions that are regulated by retinoic acid. Recent studies on alpha herpesvirus infections,^86^^,^^87^ such as HSV-1 and pseudorabies virus, found that these viruses suppress RA synthesis by increasing expression of retinol reductase 3 (DHRS3). This enzyme converts retinaldehyde back to retinol, a process dependent on virus-triggered DNA damage response and histone modifications. Treatment with palovarotene protected mice from HSV-1 infection, suggesting a potential therapeutic strategy against certain viral infections.^86^ Palovarotene is also being examined in dry eye disease, since vitamin A and retinoic acids are critical for the integrity of the ocular surface^88^ (NCT04762355). Finally, studies in a rat model of volumetric skeletal muscle defects showed that palovarotene could enhance skeletal muscle healing and neuromuscular strength.^89^

## Conclusions

The development of palovarotene as the first ever approved treatment for FOP underscores the power of basic science studies that provide essential insights into molecular and cellular mechanisms, ultimately inspiring new therapies (Figure 4). Although palovarotene can, at best, reduce new HO formation in FOP only partially, and is also associated with significant side effects and potential toxicities in patients, the palovarotene development process represents a critical first step for slowing the devastating disease progression of FOP. In addition, the seminal studies on palovarotene and the FOP NHS studies revealed critical shortcomings in the knowledge about FOP—including whether the observed negative values of HO in the MOVE study represents resorption of existing HO or compaction/maturation of a HO bone lesion. Since initiating therapies in pediatric patients with FOP is clearly a critical need, we continue to need research to better understand the mechanisms of palovarotene in both the therapeutic and adverse events. Thus, palovarotene serves as an exciting inspiration for additional studies to clarify more precisely palovarotene’s overall mode of action, spectrum of targets during FOP treatment, strategies for reducing or eliminating unwanted negative side effects, and testing of palovarotene in combination with other potential therapies being considered for FOP that may target other steps in the bone formation pathway.

## Data Availability

Not applicable.

## Abbreviations

ACVR1, Activin A receptor type 1 gene; ALK2, Activin receptor-like kinase 2 protein; atRA, All-*trans*-retinoic acid.; BCT, Bone computed tomography; COPD, Chronic obstructive pulmonary disease; DHRS3, Retinol reductase 3; DSMB, Data safety monitoring board; EMA, European Medicines Agency; FDA, Food and Drug Administration; FOP, Fibrodysplasia ossificans progressiva; HME, Hereditary multiple exostoses; HO, Heterotopic ossification; IHH, Indian hedgehog; NHS, Natural history study; PK, Protein kinase; PPC, Premature physeal closure; PRV, Pseudorabies virus; RAR, Retinoic acid receptor; RARE, Retinoic acid response element; RXR, Retinoid X receptor; SARs-CoV-2, Severe acute respiratory syndrome coronavirus 2; SMAD, Suppressor of mother against decapentaplegic; TGFβ, transforming growth factor β; WBCT, Whole body computed tomography; XLH, X-linked hypophosphatemia.
